# Porous nanocomposites with enhanced intrinsic piezoresistive sensitivity for bioinspired multimodal tactile sensors

**DOI:** 10.1038/s41378-023-00630-z

**Published:** 2024-01-26

**Authors:** Jianpeng Zhang, Song Wei, Caichao Liu, Chao Shang, Zhaoqiang He, Yu Duan, Zhengchun Peng

**Affiliations:** 1 State Key Laboratory of Radio Frequency Heterogeneous Integration (Shenzhen University), School of Physics and Optoelectronic Engineering, Shenzhen University, 518060 Shenzhen, Guangdong Province P. R. China; 2 Linksense Technology Ltd., 518060 Shenzhen, Guangdong Province P. R. China

**Keywords:** Electronic properties and materials, Electrical and electronic engineering

## Abstract

In this work, we propose porous fluororubber/thermoplastic urethane nanocomposites (**PFTNs**) and explore their intrinsic piezoresistive sensitivity to pressure. Our experiments reveal that the intrinsic sensitivity of the PFTN-based sensor to pressure up to 10 kPa increases up to 900% compared to the porous thermoplastic urethane nanocomposite (**PTN**) counterpart and up to 275% compared to the porous fluororubber nanocomposite (**PFN**) counterpart. For pressures exceeding 10 kPa, the resistance-pressure relationship of PFTN follows a logarithmic function, and the sensitivity is 221% and 125% higher than that of PTN and PFN, respectively. With the excellent intrinsic sensitivity of the thick PFTN film, a single sensing unit with integrated electrode design can imitate human skin for touch detection, pressure perception and traction sensation. The sensing range of our multimodal tactile sensor reaches ~150 Pa, and it exhibits a linear fit over 97% for both normal pressure and shear force. We also demonstrate that an electronic skin, made of an array of sensing units, is capable of accurately recognizing complex tactile interactions including pinch, spread, and tweak motions.

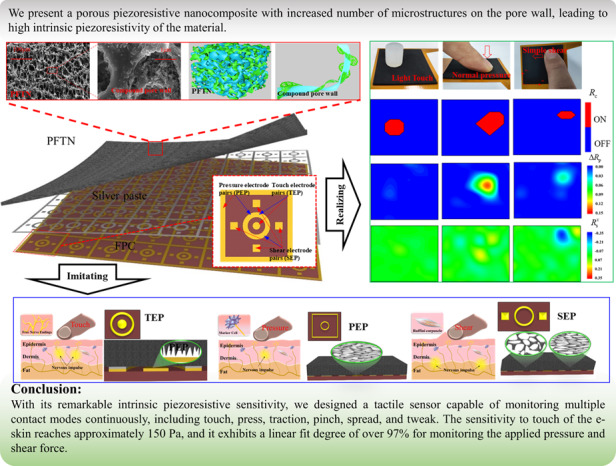

## Introduction

Electronic skin (**E-skin**) is a technology that seeks to mimic human tactile receptors through the use of advanced materials and microstructures^1–3^. By emulating the functions of the nerve free ending for touch sensation, Merkel cell for pressure sensation, and Ruffini corpuscle for traction sensation, E-skin can capture multiple contact information, including touch, press, pinch, spread, traction, tweak, and torsion. Moreover, E-skin can quantitatively monitor the motion trajectory and detect the directional trends of in-plane movements for loading points. Compared to traditional pressure arrays, E-skin offers several advantages, such as providing in-plane movement direction or trending information of force points, restoring the movement trajectory of force points by smaller lattice density, and recognizing intersecting force points with different movement directions. The underlying innovations in microstructures and advanced functional materials, specifically stretchable and lightweight porous piezoresistive elastomeric nanocomposites (**PPENs**), have been instrumental in enhancing the performance and flexibility of E-skin^4,5^. Intrinsic piezoresistive porous nanocomposites that solely rely on volumetric strain have shown remarkable potential in simulating human tactile receptors and have attracted interest from various researchers in the field^6,7^.

Piezoresistive elastomeric nanocomposites, featuring conductive nanoparticles blended into an insulating elastomeric matrix to form a conductive path, represent an attractive sensing platform for a range of applications^8–10^. The compression of these nanocomposites results in the movement of nanoparticles within the matrix and leads to an increase in interparticle contact, thereby improving the efficiency of electron transport. In particular, PPEN exhibits higher sensitivity to deformation than their solid counterparts due to the buckling or bending of pore walls. As a result, the design and fabrication of such materials has expanded to various fields, including electronics, chemistry, physics, mechanics, and materials science^11,12^. Material systems suitable for producing PPEN include thermoplastic urethanes (**TPU**), polydimethylsiloxane (**PDMS**), epoxy, and conductive fillers such as carbon nanoparticles (**CNs**), carbon nanotubes (**CNTs**), nanowires, and graphene^13^. Advanced fabrication techniques that incorporate principles of chemistry and physics have been employed to attain specific structural characteristics in electronic skin. These techniques include chemical or physical foaming, sacrificial pore-making methods, freeze drying, soaking adhesion, electroless plating, physical vapor deposition, and 3D printing, among others^6,11,14,15^. As a result, a diverse range of mechanical structures, including open-cell, closed-cell, and negative Poisson’s ratio cells, have been successfully achieved^16^. By harnessing the unique features of ultralight weight, compressibility, and breathability, these PPENs are ideal for simulating human skin in health care applications such as monitoring heart rate, breathing frequency, and pulse^14,17^, as well as for characterizing human activity patterns such as sleep, walking gait, and hand sign recognition^15,18^.

As a promising sensing platform, PPENs have garnered considerable research interest from various scientific disciplines due to their unique properties. A range of theoretical models and empirical equations have been proposed to describe their mechanical behaviors and electrical response, guiding the development of highly sensitive materials^19,20^. For example, some reports have identified pore closure as a key determinant of piezoresistive performance and have developed hierarchical PPENs to increase pore closure upon compression^21,22^. Negative Poisson’s ratio PPENs have also been achieved based on general effective media theory to demonstrate high matter density per unit volume and a more conductive path when compressed^23–26^. Internal contact resistance within these materials has been recognized as a critical factor affecting piezoresistive performance, inspiring the fabrication of multilayered PPENs with rough interfaces that increase piezoresistive sensitivity to deformation^27,28^. Researchers have also harnessed the high contact resistance at steep and sharp surfaces of these materials to attain extreme piezoresistive sensitivity for pressure monitoring^29–31^. Nevertheless, other investigations have suggested that changes in resistance may arise from the bending or buckling of pore walls, which alters the distance between conductive nanoparticles within them^32^. To enhance piezoresistive sensitivity to deformation, recent research has explored increasing the slenderness ratio of pore walls and incorporating more micropores within them using mechanical beam models and tunnel effect theory.

This study reports the development of a new porous nanocomposite, defined as porous fluororubber-thermoplastic urethane nanocomposites (**PFTNs)**, with high intrinsic piezoresistive sensitivity based on the theoretical guide outlined above. PFTNs were fabricated using a multiphase physical mixing process and the sacrifice method to achieve a homogeneous pore structure. In this approach, two different polymer materials, fluororubber (**FKM**) and thermoplastic polyurethane (**TPU**), were blended together as the matrix of the PFTN, while inorganic salt pore-making particles with uniform sizes were employed to create a porous structure with uniform pore size distribution. CNs served as conductive fillers and modified reinforcing fillers within the matrix. The high intrinsic piezoresistive sensitivity of the newly developed PFTN is attributed to two key factors: the presence of micropores distributed throughout the FKM, which arise from solvent evaporation and agglomeration of nonlinear polymer molecular chains, and the supporting role of the high-resilience linear polymer TPU, which helps to form a pore wall with a large slenderness ratio. Compared to other methods that can realize similar structures, such as 3D printing technology, this fabrication approach is cheaper, easier to process, and more readily mass-produced. By leveraging the high intrinsic piezoresistive performance of PFTNs, a multimodal E-skin mimicking different human tactile receptors was fabricated, which only required integrated electrodes, silver paste, and PFTNs. The E-skin developed in this study can capture and quantify various tactile stimuli, such as touch, pressure force, and traction force, and identify complex tactile interactions, including pinch, spread, tweak, and torsion. This study proposes a new approach to preparing PPEN with high intrinsic piezoresistive sensitivity, and extends the application of PPEN-based sensors.

## Results and discussion

### Preparation and characterization of the PFTNs

**Fig. 1:**
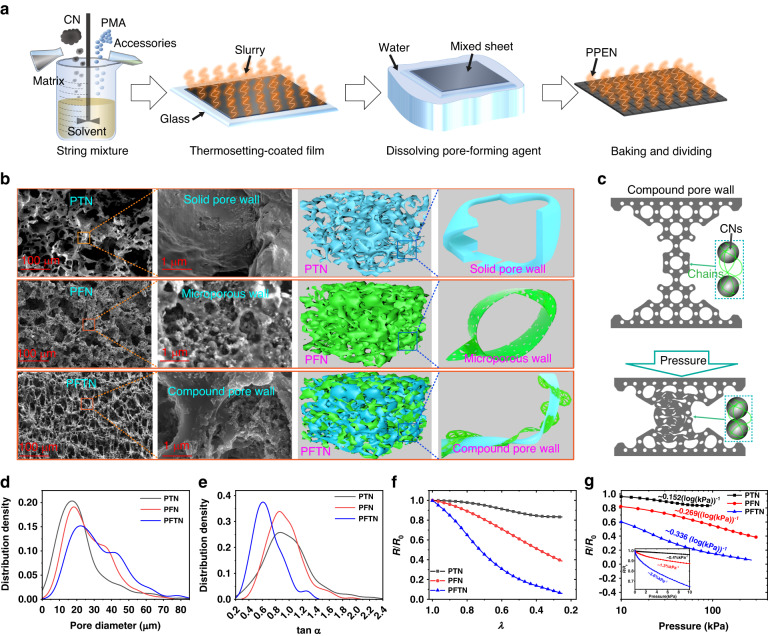
Design, preparation, microstructures, and piezoresistive characteristics of piezoresistive porous nanocomposites (PPNs). **a** Schematic diagram of the fabrication procedure of PPNs as piezoresistive sensors. **b** Scanning electron microscopy (SEM) images and schematic illustrations of the microstructures of three distinct types of PPNs—PTN (left), PFN (middle), and PFTN (right)—all containing 15 wt% CN and 750 wt% usage of sacrificial PMA (NaCl fine particles ~30 μm). **c** Schematic diagram of intrinsic piezoresistive properties of high sensitivity due to microporous wall deformation. Comparisons of pore characteristics distribution (pore diameter distribution (**d**) and pore length-width ratio (**e**)) among these PPNs, including PTN (black lines), PFN (red lines) and PFTN (blue lines). Electrical resistance-compressibility curves (**f**) and electrical resistance-pressure curves (**g**) of intrinsic piezoresistive behavior of each PPN type measured by four-point probe method

Figure 1a presents a schematic of a straightforward fabrication process consisting of four critical stages: string mixtures, thermosetting coated film, dissolving pore-forming agent, and baking and dividing. The initial phase involves the preparation of a substrate precursor fluid that incorporates several components, such as TPU dissolved into dimethylsulfoxide (**DMSO**), FKM dissolved into ethyl acetate (**EA**), CNs mixed with EA by ultrasound as conductive fillers, NaCl pore-making agent (**PMA**) ground to a particle size of 400 mesh using a planet-type grinding machine, and vulcanizing aid. The mixture is then uniformly coated onto a glass substrate and solidified in an air oven. Later, the solidified films are dissolved in purified water to remove the sacrificial pore-forming agent, after which they are baked and divided into target samples. Supplementary Fig. S1 illustrates the detailed process. TGA experiments (Supplementary Fig. S2) ensure that there are no residual salt particles within the porous material.

In Fig. 1b, scanning electron microscopy (**SEM**) images of PFTN reveal its hierarchical pore structures compared to those of porous TPU-containing CNs (**PTN**) and porous FKM-containing CNs (**PFN**). SEM images of PTN exhibit tight microstructures containing pores formed solely from sacrificial pore-forming substances with comparable diameters of approximately 20 μm. PFN displays a hierarchical pore structure comprising micropores spreading over the pore wall with a size of approximately 100 nm and additional pores produced from sacrificial pore-forming substances (~20 μm). However, due to the severe creep characteristics of the FKM substrate, PFN undergoes significant shrinkage and cracking during the dissolution of the sacrificial NaCl agent. By physically blending TPU and FKM, three improvements are observed. First, the combination of TPU and FKM leads to the formation of nanopores between their molecular chains, resulting in improved tunneling effects between conductive fillers (Fig. 1c). Second, the high-resilient TPU provides support to the FKM, which resists shrinkage and cracking, leading to enhanced porosity. Last, elongated structures along the direction of thickness emerge due to the supportive behavior of more formable TPU skeletons to the severe shrinkable FKM substrates.

Previous studies have established that the piezoresistive performance of PPEN mainly depends on its pore structure^33,34^. Hierarchical pore structures and elongated structures oriented along the direction of loading have been found to provide higher piezoresistive sensitivity than other structural forms^17^. The structural characteristics of PFTNs were obtained using visual recognition analysis (ImageJ2) (Supplementary Fig. S3), which included the distribution of pore diameters in Fig. 1d and the distribution of the length-width ratio (derived using the elliptical model in ImageJ) in Fig. 1e. The PFTN exhibits a wider range of pore diameters, demonstrating its complex hierarchical pore structures in comparison to PFN or PTN. Furthermore, the length-width ratio of the TPU/FKM blend is significantly smaller than that of TPU or FKM separately, allowing it to generate higher sensitivity. To estimate the intrinsic piezoresistive sensitivity of PPENs, four wires, i.e., four-point probe method, were employed (Supplementary Fig. S4) to eliminate the influence of contact resistance. We define ***λ*** as the compression ratio (the ratio of the thickness after compression to the initial thickness). The experimental results in Fig. 1f indicate that the sensitivity to deformation of the PFTN is up to 640% higher than that of the PTN and 200% higher than that of the PFN. The intrinsic resistance sensitivity of the PFTN with respect to pressure, as revealed in Fig. 1g, exhibits two characteristic segments. Specifically, for pressures below 10 kPa, the pressure-resistance relationship displays clear linear properties, and PFTN exhibits a significantly higher sensitivity of up to 900% and 275% compared to PTN and PFN, respectively. For pressures exceeding 10 kPa, the pressure-resistance relationship follows a logarithmic function, and the sensitivity of PFTN to the logarithm of pressure is observed to be 221% and 125% higher than that of PTN and PFN, respectively. This logarithmic relationship in PFTN with respect to the variation of pressure and resistance enables accurate simulation of the physical sensor intensity, consistent with Weber-Fechner’s law^35^, which states that the logarithmic values of the physical stimulus magnitude are directly proportional to the physical intensity of sensing. In turn, the change in intrinsic resistance can be directly and linearly reflected to quantify the intensity of pressure perception, which enables better emulation of the functions of human skin. The sensitivity increases with the mass fraction of FKM due to the increase in micropores, reaching a maximum at a mass ratio of 3:7 between TPU and FKM, after which the sensitivity decreases due to the shrinkage of FKM for higher mass fractions. Additional details can be found in Supplementary Fig. S5.

### Microstructure and piezoresistive properties of the PFTNs

**Fig. 2:**
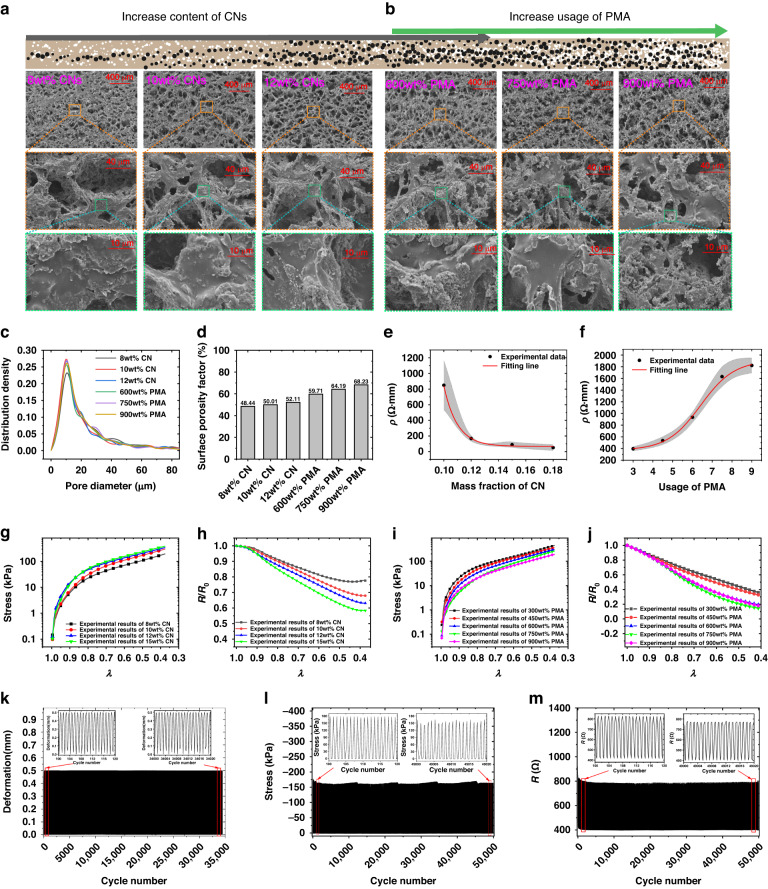
Electrical and mechanical characteristics of PFTN. SEM images of PFTNs with different CN contents (450 wt% usage of PMA) (**a**) and different usages of sacrificial PMA (15 wt% CN) (**b**), showing interconnected open-pore structures (top), single pore structural features (middle) and pore wall structures (bottom). **c** Pore diameter distribution curves of various PFTNs with different CN contents (450 wt% usage of PMA) and different usages of sacrificial PMA (15 wt% CN). **d** The surface porosity factors of these PFTNs. The original resistance of these PFTNs depending on different CN contents (450 wt% usage of PMA) (**e**) and different usage of PMA (15 wt% CN) (**f**). Stress-compressibility curves (**g**, **i**) and piezoresistive sensitivity curves (**h**, **j**) of these PFTNs with different CN contents (450 wt% usage of PMA) and different usage of PMA (15 wt% CN). Cyclic loading characteristics of a PFTN sample (750 wt% SN, 15 wt% CN), including linear loading deformation (1 Hz linear loading) (**k**), stress response (**l**), and intrinsic resistance response (**m**)

In this section, we explore the impact of the key component contents (CN and PMA) on the electrical characteristics and mechanical properties of PFTNs. The SEM images of PFTNs fabricated using different mass fractions of CN and PMA are presented in Fig. 2a and b, respectively. The three SEM images at the top of Fig. 2a demonstrate an evident trend that increasing the mass fraction of CN leads to higher porosity levels in PFTN due to the increased viscosity of the precursor slurry, which impedes shrinkage of porous structures. In contrast, the three images at the top of Fig. 2b reveal that increasing the usage of PMA leads to greater porosity and irregular pores formed by agglomerated salt clusters. The middle images in Fig. 2a, b support that higher porosity levels can decrease the effective thickness of the pore wall, while the bottom images in Fig. 2a, b indicate that the structure of the pore wall is not significantly affected by porosity. To quantitatively analyze the impact of CN and PMA on PFTN pore structures, we conducted a statistical analysis using ImageJ2 (Fig. 2c, d). The results show a similar distribution of pore diameters for different PFTN samples, with increased porosity observed with higher mass fractions of CN and greater usage of PMA. These findings highlight the critical roles that CN and PMA play in shaping PFTN pore structures and the importance of controlling their concentrations for desired electrical and mechanical properties.

Figure 2e, f illustrates the electrical characteristics of PFTNs with varying CN content and PMA usage, respectively. The electrical resistivity, which is based on a percolating network, experiences a sharp drop at a specific fraction of CN. Increasing the usage of PMA decreases the conductive path within the PFTN, causing an increase in the electrical resistivity. Many theoretical models have been established to predict these electrical resistivities^32^. In addition, Fig. 2g–j presents the piezoresistive performance (stress‒strain curves and resistance-strain curves) that was studied. The stress‒strain curve is in accordance with the highly compressed hyperelastic model proposed by Storaker^36^, with key parameters provided in Supplementary Table S1. Due to the reinforcing effect of carbon on the polyimide, the stress increases with increasing carbon content^37^. According to the effective medium theory, the stress decreases as the usage of PMA increases^32^. Empirical models of mechanical response based on contents have been summarized in our previous research^14,32,38–40^. The intrinsic piezoresistive response against the strain of PFTNs indicates that increasing CN content and PMA usage can enhance sensitivity to deformation, as demonstrated by the SEM images above. Additionally, an increase in porosity can increase the intrinsic piezoresistive sensitivity. Figure 2k illustrates the load curve, portraying cyclic linear loading with a strain range spanning 0 to 50% of compressive deformation. The compression speed was maintained at 1 mm/s, and the applied compression amounted to 0.5 mm. The experimental conditions entailed subjecting the specimen to a frequency of 1 Hz, resulting in the accumulation of over 50,000 cycles. The stress response in Fig. 2l demonstrates that the PFTN possesses steady mechanical properties that resist fatigue fracture. The resistance response (Fig. 2m) demonstrates that the piezoresistive response of PFTN is robust (Supplementary Fig. S6), except for the original decrease in resistance caused by the creep characteristics of the polymer composites. The material exhibits excellent storage performance and reusability (Supplementary Fig. S7a), maintaining consistent initial impedance and piezoresistive sensitivity after being stored for 6 months at room temperature and 30 h at a high temperature of 85 degrees Celsius. Furthermore, even after 50,000 cycles of loading, the material retains its intact and uniform porous structure (Supplementary Fig. S7b).

### Bioinspired design of the PFTN-based multimodal tactile sensor

**Fig. 3:**
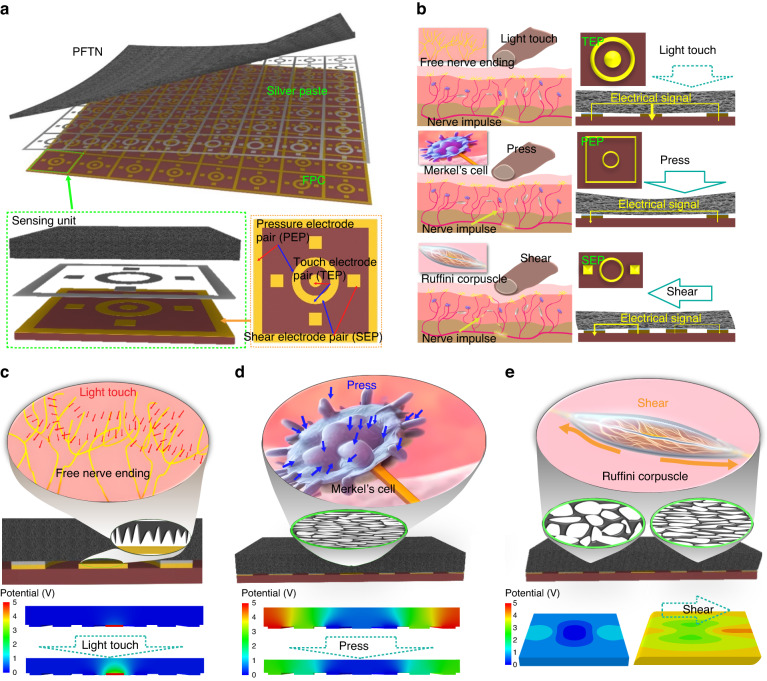
Skin-mimicking design of the E-skin and its working mechanism. **a** Schematic diagram of the E-skin comprised of an array of sensing units (left bottom) containing PFTN, silver paste, and FPC (right bottom). **b** Biomimetics of the E-skin: TEP simulating free nerve ending (top), PEP simulating marker cell (middle), and SEP simulating Ruffini corpuscle (bottom). Schematic diagram and FEA results of TEP for touch detection (**c**), PEP for pressure monitoring (**d**), and SEPs for shear monitoring (**e**)

A schematic diagram of the E-skin is presented in Fig. 3a, which comprises a PFTN layer acting as the sensitive layer, silver paste as a bonding layer to conduct electrical signals and minimize the contact resistance (Supplementary Fig. S8), and a flexible printed circuit (**FPC**) layer to connect to the data acquisition system. As shown in the bottom left of Fig. 3a, every unit within the E-skin serves as a comprehensive sensory unit that is capable of detecting a wide range of external stimuli, including touching, pressure, and traction. Each sensing unit has an FPC consisting of seven electrodes, including a common ring electrode, a circular electrode for touch detection (**TEP**), four square electrodes for traction perception (**SEP**), and a circumjacent quadra electrode for pressure perception (**PEP**) with reduced in-plane inhomogeneity. Figure 3b demonstrates the biomimetic mechanism of the E-skin, in which the electrical signal generated is similar to a nervous impulse, due to responding to external stimuli and transmitting signals to the processing unit. The sharp and steep contact interface between the PFTN and TEP in the E-skin is analogous to reticula-free nerve endings that rely on their dense distribution to achieve highly sensitive detection. Depending on the PEP capturing bulk resistance of the PFTN, every pore acts as a Merkel cell to monitor compression deformation by their volumetric strain changing the conductive performance. Additionally, four SEPs can recognize the inhomogeneity of the compressive state of the E-skin units, thus gaining the traction sensation similar to the Ruffini corpuscles.

To recreate tactile receptors within the skin, previous research has proposed three types of electronic skin with distinct features (Supplementary Fig. S13). The first type utilizes advanced functional materials, such as ion-conductive materials, to detect various signals, such as temperature and strain^41^. However, previous studies have only reported the distribution of strain, lacking information on the direction of stress. The second type employs a multilayered structure to monitor multidimensional forces by analyzing the differences in the stress/strain distributions of each layer^42^. Nevertheless, fabrication complexity remains a significant challenge. The third type measures multidimensional stress fields by using high-density array pressure sensors, which are commonly based on micro/nanofabrication technologies, presenting complex structures and poor reliability^43,44^. Here, we proposed a novel sensor utilizing highly porous materials with an outstanding intrinsic resistance sensitivity, theoretically enabling continuous measurement of tangential forces. Furthermore, the sensor fabrication process is straightforward. In our design, the measurement of three-dimensional forces was achieved by a single sensing unit. By using six integrated electrode pairs on a single sensing unit (Fig. 3a), we can measure three different modes of force interaction, including touch, pressing, and shear with high resolution while reducing the number of electrodes by approximately 50%. Additionally, the three-dimensional force sensing unit (~5 mm) is the smallest known size using piezoresistive properties. Figure 3c–e provides a detailed bionic design of the tactile perception mechanism through both structural representation and finite element analysis (**FEA**). The high sensitivity of touch is attributed to the precise design of the silver paste step between the common ring electrode and the circular electrode, which creates a critical contact state between the PFTN and the circular electrode. The microporous structure between the PFTN and TEP can effectively mimic the free nerve endings in the epidermis of the skin and capture subtle tactile information. These microporous structures can simulate the function of free nerve endings because they provide a large surface area to increase contact with the surrounding environment while maintaining sufficient sensitivity to detect light touch and pressure distribution. This critical contact state is highlighted in the schematic diagram of the local magnification in Fig. 3c, which shows that when the pressure exceeds a certain threshold, the contact resistance decreases significantly, as shown at the bottom of Fig. 3c. The modification of pores in response to pressure stimuli has the capability of mimicking the behavior of Merkel cells present in the dermis layer of the skin. The rich pore structure provides a platform for reproducing the abundant protrusions found in the skin. In Fig. 3d, an FEA model is established to show the intuitive variation characteristics of the piezoresistive performance based on the experimental results and fitting results of PFTNs containing 15 wt% CN and 750 wt% PMA in Fig. 2i. When the PFTN is compressed, the pore wall bends and buckles, as shown in the schematic representation of local magnification in Fig. 3d, thereby improving the conductance of the conductive path between conductive fillers. Additionally, Ruffini corpuscles specialize in sensing tension and exhibit high sensitivity to nonuniform skin pressure. Analogous to Ruffini corpuscles, such nonuniform pressure can also give rise to varying patterns of electrical resistance distribution. If a shear is loaded onto the top surface of the PFTN, nonuniform compression deformation occurs due to the adhesive fixation effect of the common ring electrodes. This dynamic leads to greater compression deformation in the shear direction than in the negative direction (Fig. 3e), creating a nonuniform potential distribution in the shear direction, as shown at the bottom of Fig. 3e.

### Characterization of the PFTN-based tactile sensing unit

**Fig. 4:**
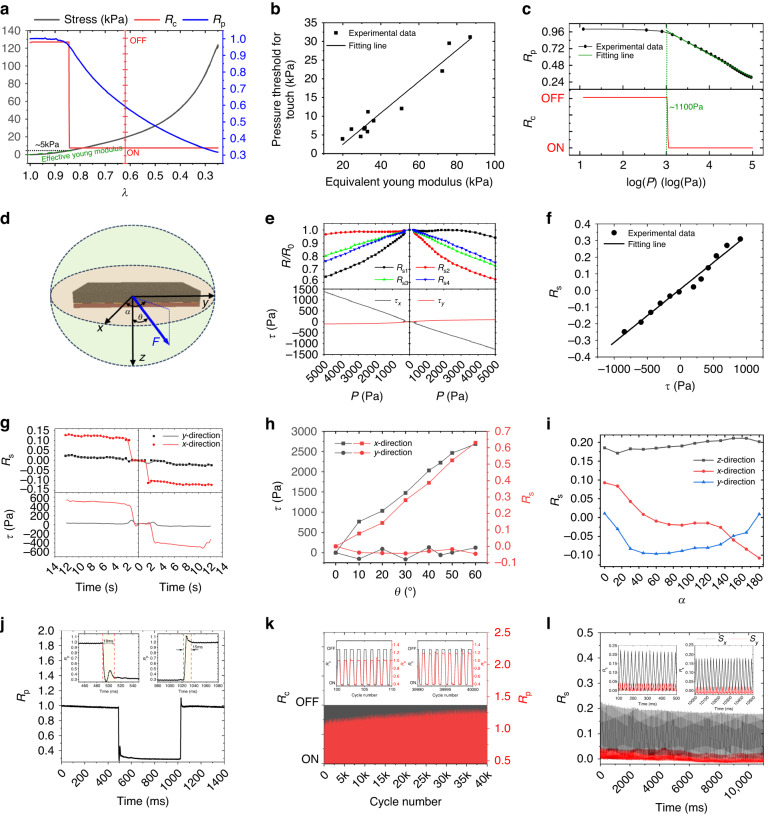
Characterization of the E-skin unit. **a** Piezoresistive response of the E-skin unit, including stress-compressibility curves (black line), resistance response of TEP (red line) and PEP (blue line), mutational site (~5 kPa) of red line donating touch threshold (black dots), and equivalent Young’s modulus (green dot) of PFTN for touch threshold. **b** Linear correlation between the touch threshold and the equivalent Young’s modulus determined by different PFTNs. **c** Intrinsic resistance change (*R*_p_) between PEP and its correlation with contact resistance (*R*_c_) between TEPs. **d** Spherical coordinate frame located on the E-skin unit. **e** Resistance change between different TEPs, including *R*_s1_ (positive *x*-direction), *R*_s2_ (negative *x*-direction), *R*_s3_ (positive *y*-direction) and *R*_s4_ (negative *y*-direction), and shears (*τ*_x_ and *τ*_y_) from the commercial sensor. **f** Linear relationship between *R*_s_ of the E-skin unit and the shear of the commercial sensor. **g** *R*_s_ (top) and the shear (bottom) in the *x*-direction and *y*-direction changing with the movements along the *x*-axis (*θ* = 90° and *α* = 0°). **h** Comparison of *R*_s_ and the shear in the *x*-direction and *y*-direction under different angles (*θ*) along the *z*-axis. **i** *R*_s_ and *R*_p_ changing with in-plane angle *α* (*θ* = 90°). **j** Response time of *R*_p_ under 75% compressed deformation. **k** Cyclic test of the intrinsic resistance change (R_p_) (black line) and the contact resistance (*R*_c_) (red line) under a 1 Hz rectangular wave (50% duty). **l** Cyclic test of *R*_s_ under 1 Hz linear loading with normal pressure (~20 kPa)

The optimized PFTN, consisting of 15 wt% CN, TPU and FKM in a ratio of 3:7 and 750 wt% PMA, is utilized as a sensing material to develop multimodal tactile sensing unit, measuring 10 × 10 mm with excellent sensitivity to touch detection, pressure perception, and traction perception. The touch and pressing response curves along with compressed compressibility ($\lambda =H/{H}_{0}$, where *H* and *H*_0_ are the compressed thickness and original thickness, respectively) are shown in Fig. 4a. ${R}_{{\rm{c}}}$ represents the on-off characteristics observed between TEPs, whereby off of ${R}_{{\rm{c}}}$ refers to the resistance measured over the range of the acquisition circuit (approximately 10 MΩ). On the other hand, ${R}_{{\rm{p}}}$ represents the inherent resistance variation. The application of force exceeding a threshold of approximately 5 kPa (as designated by black dots in Fig. 4a) leads to the collapse of the gap between the central circular electrode and PFTN, resulting in the formation of a closed circuit denoted by the red line in Fig. 4a. Compared to traditional silicon-based or screen-printing technology pressure sensors^45,46^, sensors based on PFTNs demonstrate higher sensitivity. In contrast, when compared to contact structures formed by porous micro/nanostructures that provide high sensitivity^47^, this sensor exhibits relatively lower sensitivity but higher stability. The effective Young’s modulus of the PFTN layer is estimated to be approximately 20 kPa (green dots in Fig. 4a). Figure 4b demonstrates that the threshold can be controlled by modifying the effective Young’s modulus, as evidenced by the linear relationship between the threshold and the effective Young’s modulus. The high responsiveness and sensitivity of the pressure sensor are attributed to the micro/nanoporous interface between the porous material and the electrode. This interface allows for deformation upon contact, leading to a significantly heightened response to pressure. The sensor’s performance approaches that of a high-sensitivity pressure sensor based on micro/nanostructures^46–48^. Moreover, as the porosity increases, the interface porosity structure becomes more pronounced, and the material modulus decreases, further enhancing the sensor’s sensitivity to changes in pressure. Electrical resistance-pressure curves of the PFTN, depicted in Fig. 4c, follow a linear function with the natural logarithm of pressure as per Stroker’s model, which exhibits nonlinear stress‒strain relationships that satisfy an exponential function. When the pressure exceeds the contact threshold between the TEP and PFTN, a linear correlation is observed between the change in electrical resistance and the natural logarithm of pressure. This correlation is expressed mathematically as

1 $$P=1100{{\rm{e}}}^{2.71(1-{R}_{{\rm{p}}}^{{\prime} })}({\rm{Pa}}),$$

where ${R}_{{\rm{p}}}^{{\prime} }=R/{R}_{0}^{{\prime} }$ represents the intrinsic resistance change, *P* denotes the applied pressure, and ${R}_{0}^{{\prime} }$ corresponds to the electrical resistance at the contact threshold pressure. The proposed multidimensional force sensor based on porous structures exhibits higher sensitivity than known multidimensional force sensors, such as electromagnetic and silicon-based piezoresistive sensors, which are known for their highly linear shear force sensing capabilities^46,49^. The logarithmic relationship between pressure and resistance exhibited by the PFTN can better simulate the pressure-sensing function of human skin and achieve a linear change in the resistance change signal of the sensor with the perceived external pressure intensity by the human psyche. according to Weber-Fechner law^35^. Thus, the PFTN’s sensitivity to pressure can more accurately reflect the human somatosensory response to various pressure levels. Notably, the magnitude of *R*^2^, which quantifies the goodness-of-fit for the linear model, exceeds 99%, thereby indicating a very tight association between the variables of interest in this particular scenario. The sensitivity of the sensor to pressure depends on two main factors: increasing the dosage of PMA can increase the porosity of the material, thereby reducing its hardness and increasing its deformation under force. Additionally, the increased porosity enhances the material’s internal microstructure, including its nanoscale features, which increases its sensitivity to changes in resistance due to deformation. Notably, our results indicate that increasing the usage of PMA can enhance sensitivity to deformation, decrease the effective modulus, and increase sensitivity to pressure (Fig. 2i, j).

To assess the shear response characteristics of the unit, a spherical coordinate frame is established, as depicted in Fig. 4d, to differentiate between diverse shear deformation cases. *θ* and *α* variables represent the loading angle with the normal direction out of plane (*z*-axis) and the horizontal direction in the plane (*x*-axis), respectively. Figure 4e demonstrates the variations in the four resistances in the orthogonal direction with increasing loading amplitude, as well as the response of the commercial three-dimensional force sensor under loading angles of *θ* = 45° and *α* = 0 (Supplementary Fig. S9a). Notably, the resistances in the direction of shear deformation exhibited significant differences, indicating that shear deformation occurred. Conversely, the resistances in the orthogonal direction exhibited similar trends due to the same compressed deformation. The following proposed Equation 2 describes shear deformation by utilizing the change in electrical resistance between SEPs. The equation is defined as follows:

2 $$\left\{\begin{array}{c}{R}_{{\rm{s}}}^{x}=\frac{{R}_{{\rm{s}}1}}{{R}_{{\rm{s}}1}^{0}}-\frac{{R}_{{\rm{s3}}}}{{R}_{{\rm{s3}}}^{0}}\\ {R}_{{\rm{s}}}^{y}=\frac{{R}_{{\rm{s2}}}}{{R}_{{\rm{s2}}}^{0}}-\frac{{R}_{{\rm{s4}}}}{{R}_{{\rm{s4}}}^{0}}\end{array}.\right.$$

where, ${R}_{s}^{x}\,{\rm{and}}\,{R}_{s}^{y}$ refers to the change in the SEP electrical resistance caused by shear deformation. ${R}_{s1},\,{R}_{s2},\,{R}_{s3}\,{\rm{and}}\,{R}_{s4}$ correspond to the electrical resistance values of SEP in the positive and negative directions of the *x*-axis and the positive and negative directions of the *y*-axis, respectively. Additionally, ${R}_{s1}^{0},\,{R}_{s2}^{0},\,{R}_{s3}^{0}\,{\rm{and}}\,{R}_{s4}^{0}$ represents the initial resistance value of SEP.

These findings, as illustrated in Fig. 4f, demonstrate a significant linear correlation between shear stress (τ) and the value obtained from Equation 2. This relationship is appropriately described by an equation shown as:

3 $$\tau =121.07{R}_{s}.$$

The computed linear fitting degree exceeds 97%, indicating strong predictability of the model. In addition, Fig. 4g depicts the shear response of the tactile sensing unit and commercial three-dimensional force sensors to shear loading exerted in the *x*-direction under normal pressure (~20 kPa) (as demonstrated in Supplementary Fig. S10c). The results indicate that our sensing unit exhibits good consistency with commercial sensors in tangential force monitoring and other aspects. Furthermore, the resolution of loading angles with respect to the *x*-axis (*α*) and *z*-axis (*θ*) is studied. The shear response of a unit under varying loading angles *θ* but consistent pressure (~4 kPa) is illustrated in Fig. 4h, with further analysis of changes in SEP electrical resistance presented in Supplementary Fig. S10a, b. Impressively, the observed response exhibits a high degree of similarity to that obtained using commercially available three-dimensional force sensors, thereby emphasizing the precision of the unit for monitoring shear stress. Furthermore, by changing the shear angle *α*, three-dimensional force response curves are plotted, as shown in Fig. 4i. Notably, compared to the cosine curve of shear in the *x*-direction, the sine curve of shear in the *y*-direction, and the settled curve of pressure from the commercial sensors (as shown in Supplementary Fig. S10d), the responses of the sensing unit closely follow a similar pattern of changes. Compared to rigid piezoresistive materials, the use of flexible porous piezoresistive materials offers distinct advantages in terms of pressure sensing. Due to their lower modulus, these materials exhibit heightened sensitivity, resulting in superior pressure resolution. Experimental analysis revealed that the accuracy of touch recognition reached approximately 150 Pa (Supplementary Fig. S11b). Moreover, the pressure perception accuracy was measured at an impressive 150 Pa (Supplementary Fig. S11c), showcasing the material’s precision in discerning subtle variations in pressure. Similarly, traction force recognition demonstrated a commendable precision of 200 Pa (Supplementary Fig. S11f). These findings highlight the remarkable capabilities of flexible porous piezoresistive materials in achieving high-precision pressure sensing and touch recognition.

The practicality of the multimodal sensing unit in terms of response speed and cyclic robustness is also studied. The recovery of *R*_p_ is dependent on the recovery of the deformation of the entire sensor, resulting in a longer response time compared to *R*_c_ and *R*_s_. This response time falls within the range of 10-20 ms at 75% strain, as shown in Fig. 4j. With the high elastic modulus of TPU material, PFTN material possesses remarkable elasticity, surpassing that of other flexible resistive materials^45,50^, thereby achieving superior response speed as compared to other designs. The cyclic loading features of *R*_c_ and *R*_p_ under pulse loading of the unit are demonstrated in Fig. 4k. This study reveals that the cyclic touch detection responses remain robust regardless of being ON or OFF, whereas pressure monitoring demonstrates minimal drift akin to the cyclic characteristics of intrinsic resistance. The observed drift tends to decrease as the PMA usage increases (Supplementary Fig. S6), which possibly occurs due to the porosity of PFTN decreasing the concentration of strain within its porous structure. The cyclic linear shear loading features of the unit in the *x*-direction are demonstrated in Fig. 4l, indicating the robustness of the unit for monitoring shear stress. Overall, these findings validate the precision of our sensing unit for monitoring shear stress with varying loading conditions, including that in the horizontal plane. The primary tactile information can be effectively captured using a four-key parameter vector$({R}_{{\rm{c}}},{R}_{{\rm{p}}},{R}_{{\rm{s}}}^{x},{R}_{s}^{y})$.

### Monitoring of complex tactile interactions by a PFTN-based E-skin

**Fig. 5:**
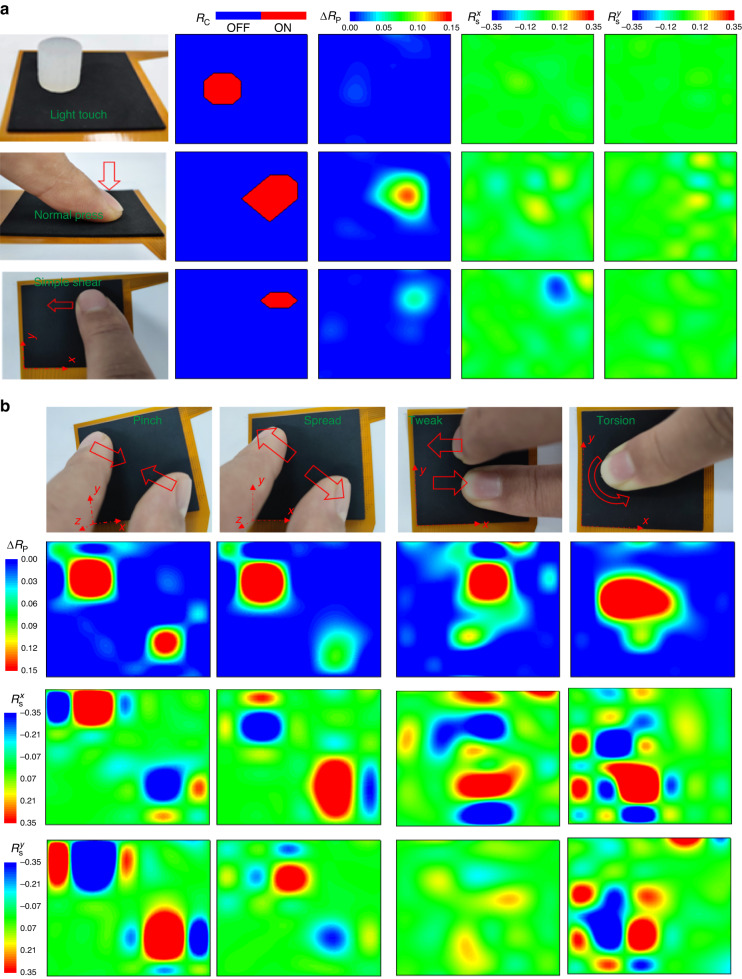
Demonstration of the capability of the multimodal E-skin. **a** Images and contours (*R*_c_, *R*_p_, *R*_s_^*x*^, *R*_s_^*y*^) of the E-skin under different loading states: light touch of soft silicone (top), normal pressure (middle) and negative *x*-directional shear (bottom). **b** Images and contours (*R*_p_, *R*_s_^*x*^, *R*_s_^*y*^) of the E-skin under different traction modes: pinch (left), spread (left middle), tweak (right middle) and torsion (right)

This section introduces an E-skin that is based on an array of the tactile sensing unit. The sensing unit of the E-skin has a size of 5 × 5 × 1 mm and exhibits similar performance to the unit presented in Section (Characterization of the PFTN-based tactile sensing unit), as shown in Supplementary Fig. S12. Compared to a multidimensional force sensor array fabricated with costly micro/nano techniques, which realize a spatial resolution of approximately 2 mm^45^, our E-skin achieves a similar spatial resolution (~5 mm) at a much lower cost. Using an array test circuit (as shown in Supplementary Fig. S14), contours of tactile information related to touch detection, pressure perception, and traction perception are obtained under various loading conditions. Our E-skin exhibits a high level of biomimicry in discerning various tactile states, including three combined states: individual touch detection (top in Fig. 5a), a combination of touch detection and pressure perception (middle in Fig. 5a), and a combination of touch detection, pressure perception, and traction perception (bottom in Fig. 5a). The threshold of the E-skin for touch sensation is defined as the contact impedance becoming less than 10 MΩ, indicating the establishment of a contact pathway between the PFTN and the electrodes (TEP). To simulate the light touch of human skin, a soft Ecoflex rob (~2.5 g, Ф15 mm × 15 mm) is used as a proxy for human fingers. When the rob is placed on the E-skin, as seen in the left image in Fig. 5a (grayscale map in Movie 1), the high sensitivity (~150 Pa) of the E-skin for detecting touch information is observed by the contour of *R*_c_. Meanwhile, contours of pressure and shear exhibit a very small response under light touch. When a human finger exerts normal pressure on the E-skin beyond the contact threshold, the tactile information obtained through the skin exhibits significant pressure information, while minute shear information is detected, as revealed by the contours of touch detection in the middle of Fig. 5a (grayscale map in Movie 2). A tangential loading trend along the negative *x*-direction is applied on the E-skin, and the contours of *R*_c_, *R*_p_, and *R*_s_ are depicted at the bottom of Fig. 5a (grayscale map in Movies 3 and 4). The contour of *R*_p_ indicates a single point of concentrated pressure information, which is similar to that of normal pressure, whereas little shear stress is observed. With a 16-bit ADC data acquisition system (Supplementary Fig. S14), the E-skin demonstrates high signal-to-noise ratios (SNRs) (Supplementary Fig. S15). Specifically, at a pressure level of 500 Pa, the SNR of the touch sensor exceeds 130, enabling precise detection of the occurrence of touch. Beyond 500 Pa, the SNR for the pressure sensor is 9, while the SNR for the shear sensor reaches 43. These findings demonstrate that our integrated tactile sensors can discern various tactile interactions of human fingers on the electronic skin.

Figure 5b demonstrates the capacity of E-skin to distinguish intricate tactile interactions, such as pinch, spread, tweak, and torsion (grayscale map in Movies 5–7). The contours of *R*_p_ under pinch, spread, and tweak movements display two distinct clusters of pressure concentration points. Moreover, the contours of ${R}_{s}^{x}$ and${R}_{s}^{y}$ exhibit values that are opposite to each other, indicating opposing tangential force directions. A comparison of the pinch and spread contours reveals similar *R*_p_ contours but opposite contours of ${R}_{s}^{x}$ and ${R}_{s}^{y}$. Under tweak loading, the contours of ${R}_{s}^{x}$ display a pair of opposing concentration points, while only a few discernible responses are observed for the${R}_{s}^{y}$ contours. Torsion loading results in the presence of a single pressure concentration point in the *R*_p_ contours, while the ${R}_{s}^{x}$ and ${R}_{s}^{y}$ contours exhibit opposing tangential force concentration points. The torque generated by the tweak and torsion loading are estimated using the following equation:

4 $$T=\mathop{\sum }\limits_{i=1}^{n}\left(\begin{array}{c}{R}_{s}^{x}\\ {R}_{s}^{y}\end{array}\right){\overrightarrow{v}}_{i},$$

where ${\overrightarrow{v}}_{i}=({x}_{i},{y}_{i})$ is the coordinate of shear forces. As per the contours illustrated in Fig. 5, the torque generated from the tweak loading is approximately 0.675N mm, whereas the torque produced by torsion loading is approximately 3.968N mm.

## Conclusion

Prior investigations on the intrinsic piezoresistivity of conductive porous nanocomposites have demonstrated that bending or buckling of the pore walls, rather than the closure of pores, is the principal factor governing the piezoresistive transduction in PPENs. Thus, increasing the number of microscopic structures on the pore walls can result in a higher intrinsic piezoresistivity. Guided by this principle, we optimized the microstructures on the pore walls of a new nanocomposite by mixing two elastomeric polymers (TPU and FKM) with the proper ratio. The PFTN-based piezoresistive sensor exhibits the highest intrinsic piezoresistive sensitivity within 10 kPa (~1.48) among known porous nanocomposites, approximately 6 times higher than that of the TPU-based sensor and two times higher than that of the FKM-based counterpart. Beyond 10 kPa, the resistance change of the PFTN to pressure follows a logarithmic relationship, which simulates the pressure-sensing function of human skin.

PFTN-based tactile sensors not only present unique advantages in monitoring volumetric strain, but the contact resistance between the porous structure and the electrode also provides an extremely high sensitivity to minute pressure. By integrating multiple electrode pairs, we successfully fabricated a flexible E-skin capable of monitoring multimodal tactile information, including touch detection, pressure perception, and traction sensation. Our E-skin design can detect the movement direction of the force with only half the number of electrodes required by a traditional 3D force sensor. The accuracy of our E-skin for measuring in-plane or out-of-plane shear was verified by a commercial 3D force transducer. Furthermore, we demonstrate that the multimodal tactile sensor can detect complex tactile interactions, such as pinch, spread, tweak, and torsion. Our E-skin can be integrated with multiple zones on a robotic body, such as the fingers, the palm and the elbow, paving the road to the development of anthropomorphic robots.

### Experimental section/methods

#### Preparation of PTN, PFN and PFTN

The sacrificial NaCl templating method is employed in the fabrication of the piezoresistive porous nanocomposites. To prepare uniform and fine NaCl particles, planetary ball milling (YXQM-20 L, MITR) is utilized to reduce the size of NaCl particles to microparticles (~30 μm). Subsequently, the target NaCl particles are extracted from the mixture using a screen machine (YC-800, XINXIANGYUCHENG) with a 400-mesh screen cloth. TPU (Elastollan® 685 A), FKM (FKM246, HUAXIASHENZHOU), and their composite are selected as the substrates for the piezoresistive porous nanocomposites. These polymers are manually cut into centimeter-sized particles and dissolved in N,N-dimethylformamide (DMF, Aladdin) solvent or ethyl acetate (EAC) solvent (99.5%, Aladdin) to form the precursor solution using a small electric blender (CS40-Pro, DLAB). Carbon nanoparticles (SUPER P Li, TIMCAL) serve as conductive fillers within the piezoresistive porous nanocomposite and are dispersed into EAC solvent (99.5%, Aladdin) using sonication (JY92-IIDN, SCIENTZ) to enhance the uniformity of dispersion. Finally, the precursor slurry and the conductive filler solution are mixed using an electric blender (10 L Double Planet Vacuum Power Mixer, DONGGUANTUOCHUAN) to form the precursor slurry. The precursor slurry is then uniformly coated onto a glass plate using a uniform coater (AT-TB-2000, SHANDONGMAITE). To eliminate the organic solvents (DMF and EAC), the glass plate was placed into an air oven (PRECISION HIGH TEMPERATURE TEST CHAMBER, DONGGUANHUITAI) at 80 °C for 6 h to achieve curing. Finally, the curing object is immersed in water for 12 h to ensure complete dissolution of NaCl microparticles. To ensure the complete dissolution of salt, a salinity meter will be utilized during the immersion process to measure the salinity of the dissolving water. Experimental evidence from TGA (Supplementary Fig. S2) confirms the thoroughness of salt dissolution when the salinity of the added pure water is below 5 psu. Upon drying, the target piezoresistive porous nanocomposites are completed.

#### Preparation and characterization of the E-skin

To fabricate the samples, the PPEN plate was polished into 1 mm thick films using a sheet machine (C420, Camoga), and their thickness was validated using a thickness gauge (YJC-6-10, YUEQING JINGCHENG). The films were then cut into the desired shape using a laser cutting machine (UV-3C, Han’s Laser). The microstructural properties of the samples were analyzed using a scanning electron microscope with field emission capabilities (Supra 55, ZEISS). The intrinsic piezoresistive properties of the samples were evaluated using a four-electrode FPC (Supplementary Fig. S4). Furthermore, a universal material testing machine (E1000, Instron) with a digital multimeter (34465A, Keysight) was used to obtain the piezoresistive performances, including stress‒strain and resistance-strain relationships. To minimize the contact resistance, silver paste (CI-1036, ECM) was used to bond the PFTN with the electrodes. The coating of silver paste was performed using screen-printing equipment (GALAXY AUTOMATIC SCREEN PRINTER, GSK) aligned with the FPC electrodes. The shear characteristics of the E-skin unit were measured using a manually adjusted tilting platform (Supplementary Fig. S9a) and a three-dimensional motion platform (Supplementary Fig. S9b). A commercial three-dimensional force transducer (FNZ-20 N, FORSENTEK) was used to evaluate the shear performance. Additionally, a 64 × 6 channel data acquisition system (Supplementary Fig. S14) based on the 16-bit, 32-channel DAQ Card (Data Acquisition Card NI-6315) and LabVIEW V2022 software were used to obtain sensor array information of the E-skin.

#### Theory and FEA analysis of intrinsic piezoresistive properties of PPEN

Based on our previous research findings, it can be concluded that the piezoresistive properties of PPENs are largely dependent on the deformation of the pore wall. Through extensive experimentation and analysis of prior research, we have proposed an empirical equation to accurately describe these properties as

5 $$\left\{\begin{array}{l}\sigma =\frac{2\mu }{\alpha \lambda }({\lambda }^{\alpha }-{J}^{-\alpha \beta })\\ \frac{\rho }{{\rho }_{0}}=\frac{1}{\lambda }(\varphi {\lambda }^{a}+(1-\varphi ){J}^{b})\end{array},\right.$$

where *λ* represents the primary compressibility of PPENs, while $J$ is a function of the volume ratio denoted by $(1-p){e}^{1/(1-p)}+p$. In this equation, *p* is the effective porosity of the PPENs, which is calculated as ${(1-SPF)}^{3/2}$. Here, *SPF* refers to the plane porosity that is determined using ImagJ2. The material parameters *μ*, *α* and *β* are essential for determining the stress response of PPENs, whereas $\varphi$, *a* and *b* are necessary for deciding the electrical resistivity changes that occur in PPENs. Using this piezoresistive model, we were able to accurately obtain these key parameters (R^2^ > 0.98), which are presented in Supplementary Tables S1 and S2. To further examine the piezoresistive behavior of PPENs and the response of the E-skin unit, we constructed FEA models utilizing a subroutine UMAT (Supplementary Fig. S16). This model consisted of 120 thousand C3D8T elements to ensure sufficient convergence of the FEA results. We incorporated a static electromechanical coupling module to derive the potential distribution and established mechanical fixed boundary conditions at the bottom with a specific displacement condition loading at the top. We also applied appropriate electrical boundary conditions to the electrodes of the FPC, as required in Fig. 3i, m.

### Supplementary information

Revised Supporting Information

Movie 1 Touch detection

Movie 2 Pressure monitoring

Movie 3 X-shear monitoring

Movie 4 Y-shear monitoring

Movie 5 Pinch monitoring

Movie 6 Spread monitoring

Movie 7 Tweak monitoring

Movie 8 Torsion monitoring
