# Supplementary material for: Porous nanocomposites with enhanced intrinsic piezoresistive sensitivity for bioinspired multimodal tactile sensors
Source: Microsyst Nanoeng. 2024 Jan 26;10:19. doi: 10.1038/s41378-023-00630-z (PMC10811241; doi:10.1038/s41378-023-00630-z)
Supplement: Supplementary file 1 — Revised Supporting Information [file 41378_2023_630_MOESM1_ESM.docx]

Supporting Information

Porous Nanocomposites with Enhanced Intrinsic Piezoresistive Sensitivity for a Highly Integrated Multimodal Tactile Sensor

Jianpeng Zhang ^a^, Song Wei ^a^, Caichao Liu ^b^, Chao Shang ^a^, Zhaoqiang He ^a^, Yu Duan ^a^, Zhengchun Peng ^a, b^ *

^a^ Center for Stretchable Electronics and NanoSensors, School of Physics and Optoelectronic Engineering, Shenzhen University, Shenzhen, Guangdong Province, P. R. China 518060

^b^ Linksense Technology Ltd., Shenzhen, Guangdong Province, P. R. China 518060

^*^ *To whom correspondence should be addressed:* *zcpeng@szu.edu.cn*


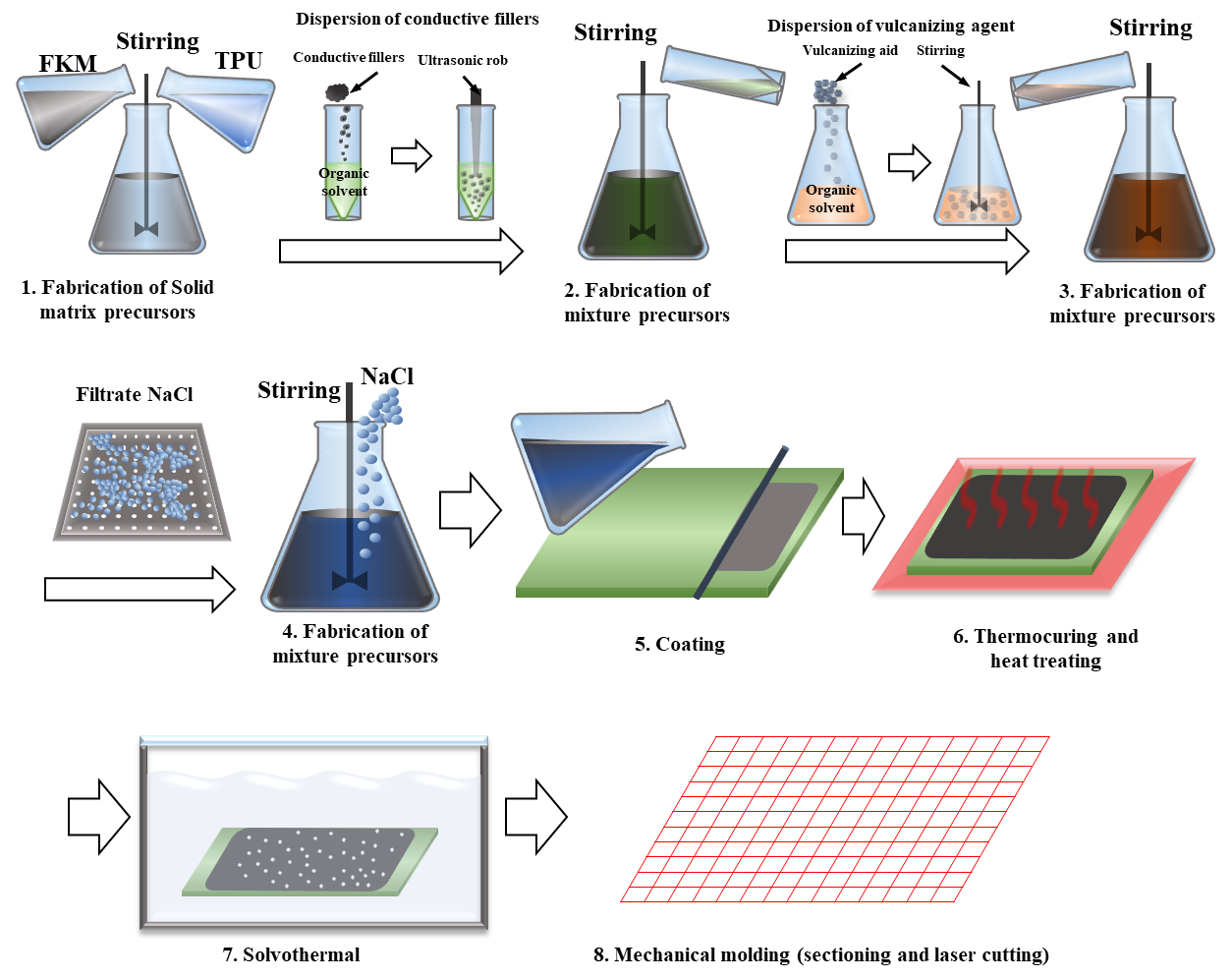


**Figure S1**. Detail fabrication procedure of highly intrinsic piezoresistive porous nanocomposites.

**Figure S2** TGA analysis curve originates from PTN containing 18wt% CN.


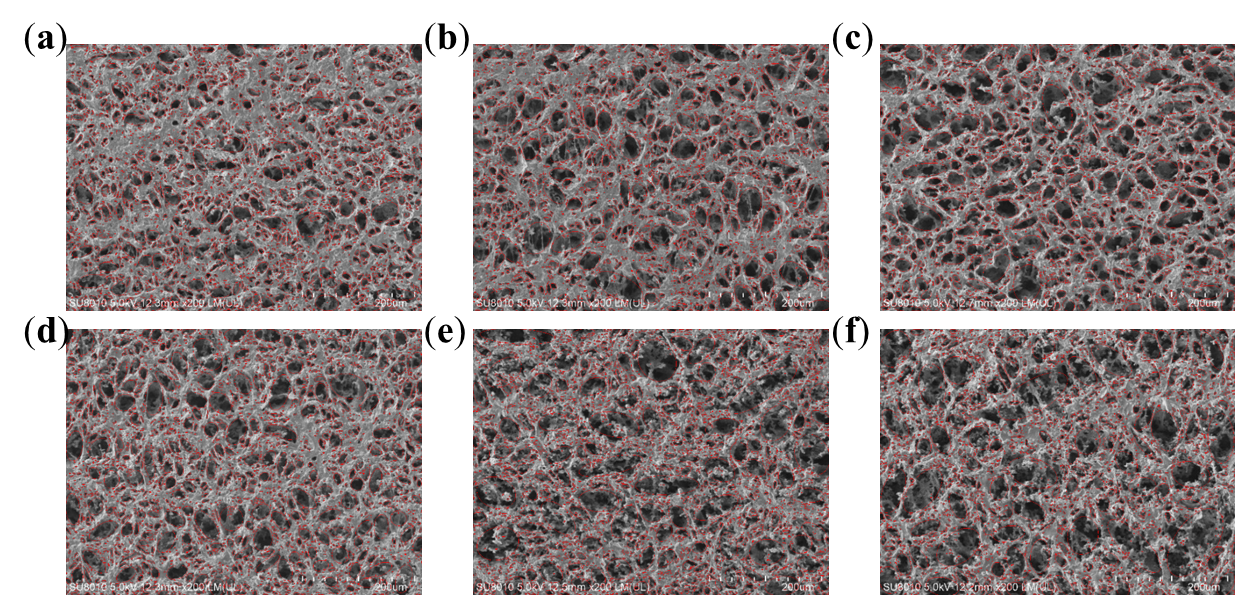


**Figure S3** Visual image recognition features of pore structure by ImagJ2. SEM images and corresponding pore structure features of PFTNs containing 400wt% usage of PMA and 8wt% CN (**a**), 10wt% CN (**b**), and 12wt% CN (**c**), as well as for PFTN containing 15wt% CN and 600wt% usage of PMA (**d**), 750wt% usage of PMA (**e**), and 900wt% usage of PMA (**f**).


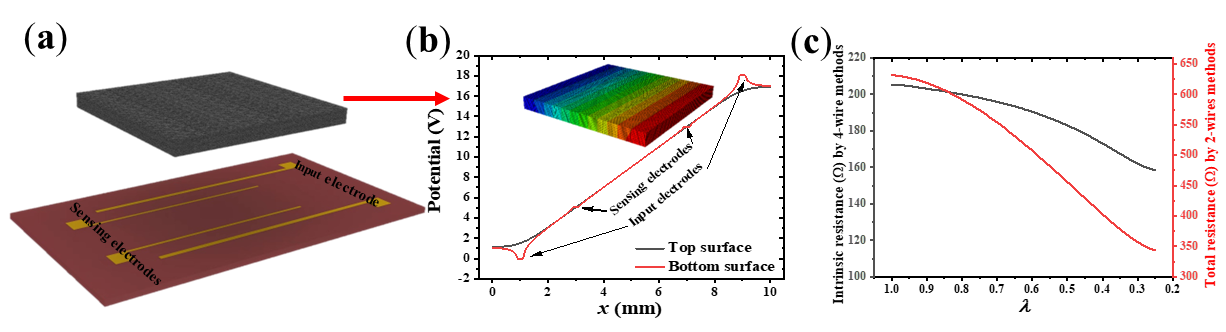


**Figure S4** Measurement of intrinsic piezoresistive properties of porous nanocomposites. (**a**) Schematic diagram of the four-electrode methods. (**b**) The potential distribution at the top and bottom of the porous nanocomposites, showing high consistency between sensing electrodes, indicating that these sensors are highly accurate in measuring the electrical resistivity of a 1mm thick porous material. (**c**) Experimental comparison between intrinsic resistance based on 4-wire method and total resistance based on 2-wire method on sensing electrodes.


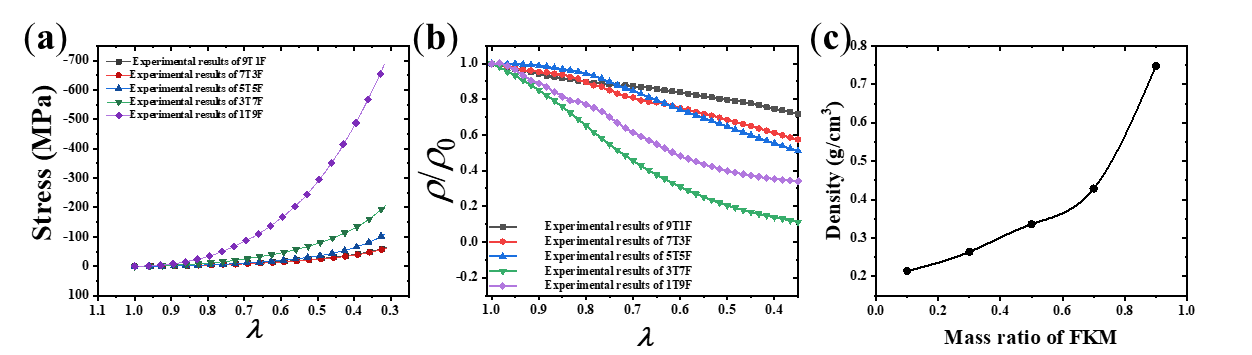


**Figure S5** Intrinsic piezoresistive characteristic of porous nanocomposites in different mass ratio between FKM and TPU, same CN content (15wt%), and same usage of PMA (750wt%). (**a**) Stress-compressibility curves of these PFTNs ("*n*T*m*F" indicates that the PFTN is composed of n parts TPU and m parts FKM). (**b**) Electrical resistivity change along with the mass ratio between FKM and TPU. (**c**) Density changing with the mass ratio between FKM and TPU.


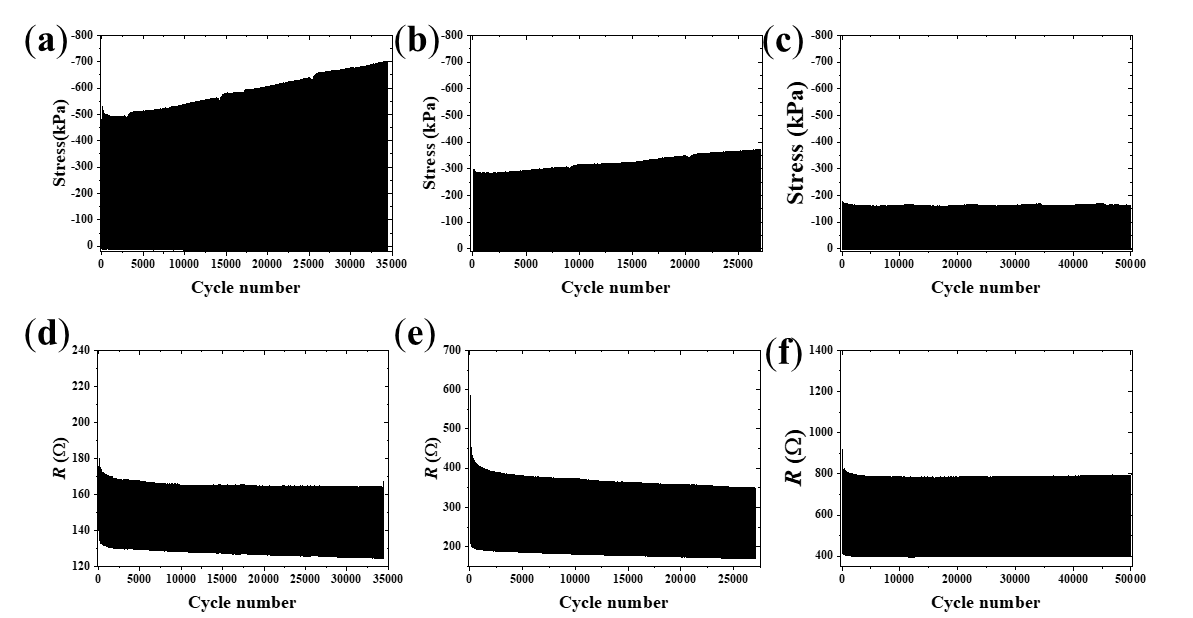


**Figure S6** Cyclic characteristics of stress and electrical resistance of PFTNs when utilizing different amounts of PMA, under 1Hz linear loading with 50% deformation. Compressed stress response of PFTNs containing 400wt% usage of PMA (**a**), 600wt% usage of PMA (**b**), and 750wt% usage of PMA (**c**) respectively, as well as the electrical resistance response of PFTNs containing 400wt% usage of PMA (**d**), 600wt% usage of PMA (**e**), and 750wt% usage of PMA (**f**).


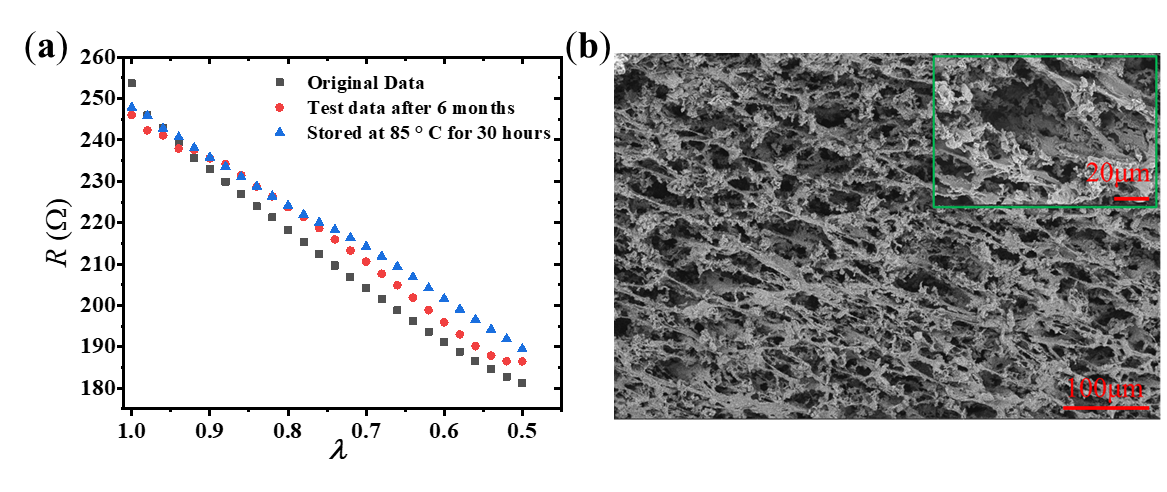


**Figure S7** Excellent storage performance and reusability of PFTN. (**a**) Piezoresistive curves of a PFTN under stored at room temperature for 6 months and at 85 ° C for 30 hours. (**b**) SEM figure of the PFTN after undergoing 50,000 cycles of loading.


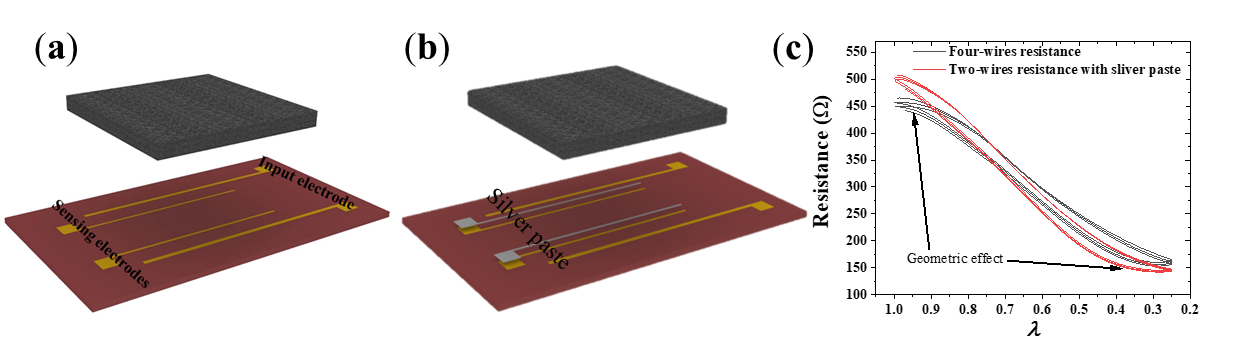


**Figure S8** Experimental evaluation of using silver paste to eliminate contact resistance. Schematic view of a typical four-wire method (**a**) and the corresponding two-wire method utilizing silver paste to make contact (**b**). (**c**) Compressed electrical resistances under three cycles of linear loading for both methods.


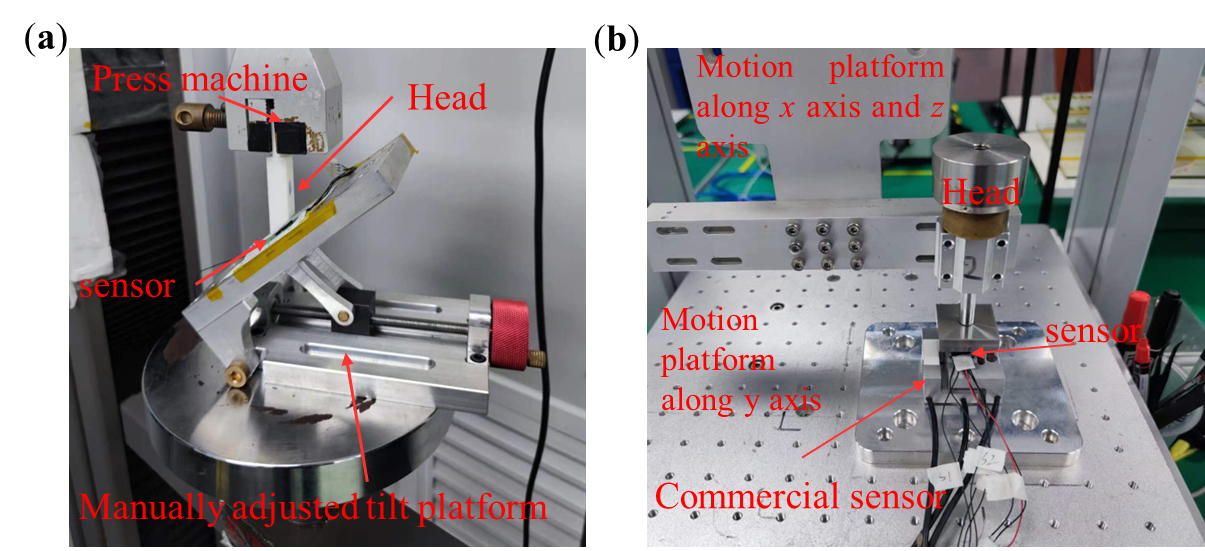


**Figure S9** Images of the different testing platforms that were utilized to measure three-dimensional forces. (a) Image of the manually adjusted tilting platform combined with a press machine. (b) Image of the three-dimensional motion platform along with a commercial sensor.


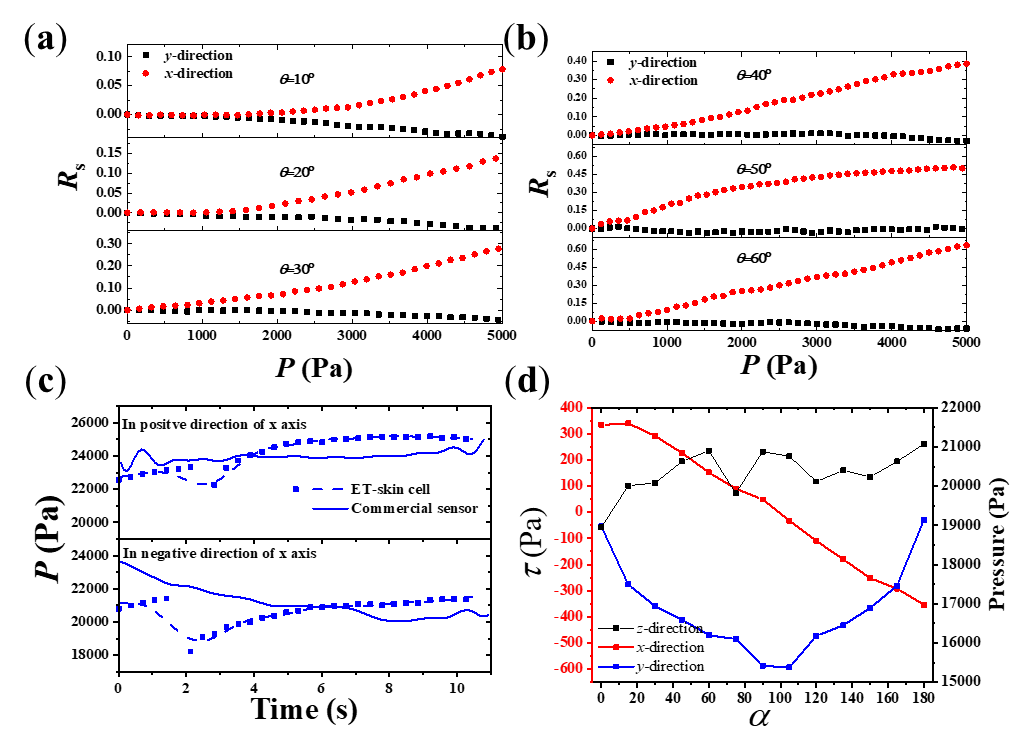


**Figure S10** Tangential force monitoring characteristics of the ET-skin cell, as well as the commercial sensor. (**a, b**) The electrical resistance change curves between TEPs along x-direction (red dots) and y-direction (black dots) under different out-plane angles with respect to z axis. (**c**) Normal pressure on the sensor applied an in-plane tangential force (θ=90°, α=0°or 180°). (d) The response of three dimensional forces of commercial senor when applied different in-plane angle *α* of tangential force (θ=90°).


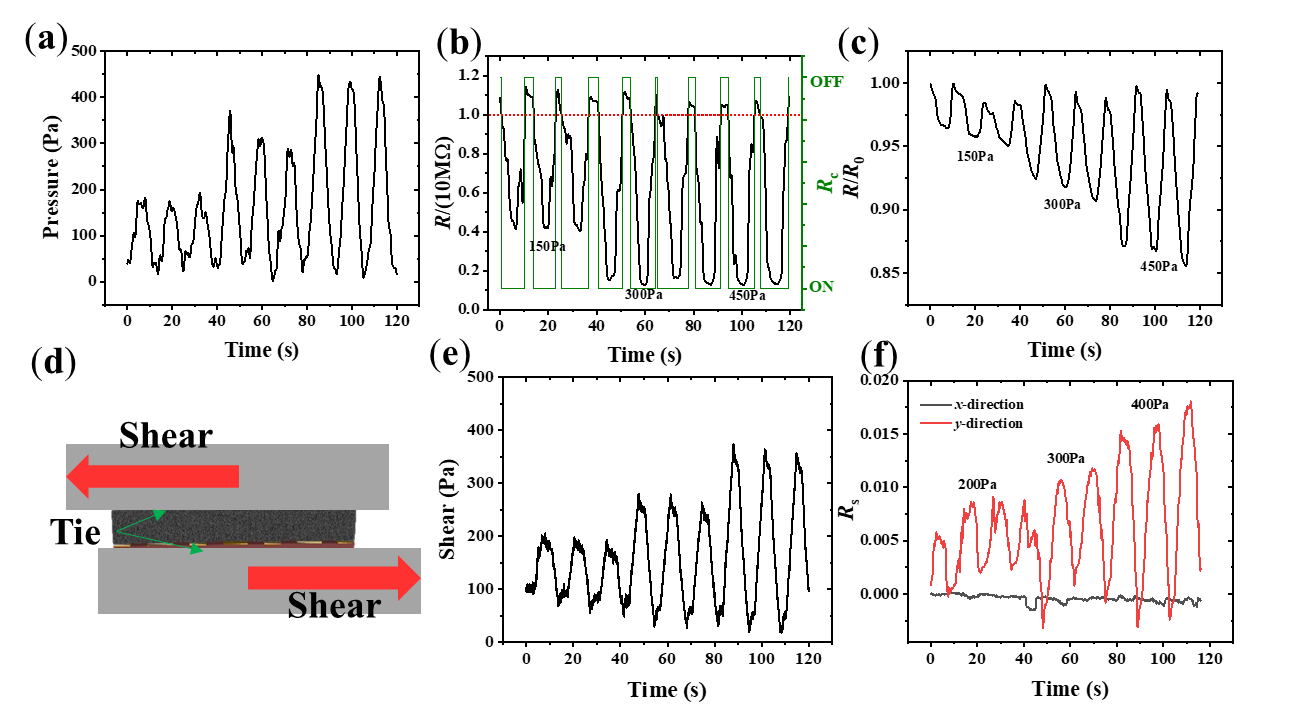


**Figure S11** Recognition accuracy of the E-skin cell. (**a**) Normal pressure curve from the compression machine. (**b**) Contact resistance curve of TEP under normal pressure. (**c**) Electrical resistance curves of PEP under normal pressure. (**d**) Tangential loading schematic diagram. (**e**) Shear curves from the compression machine. (**f**) Shear resistance response curve of the sensor.


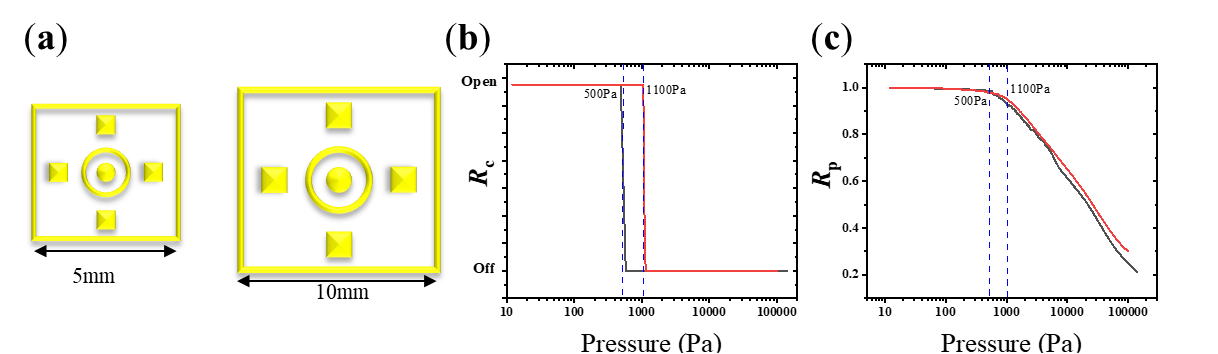


**Figure S12** Comparison of piezoresistive response of ET-skin cell containing different size electrodes. (**a**) Schematic view of electrodes with size of 10mm and 5mm. *R*_c_ curves (**b**) and *R*_p_ curves (**c**) changing with normal pressure on ET-skin cell containing both electrodes.


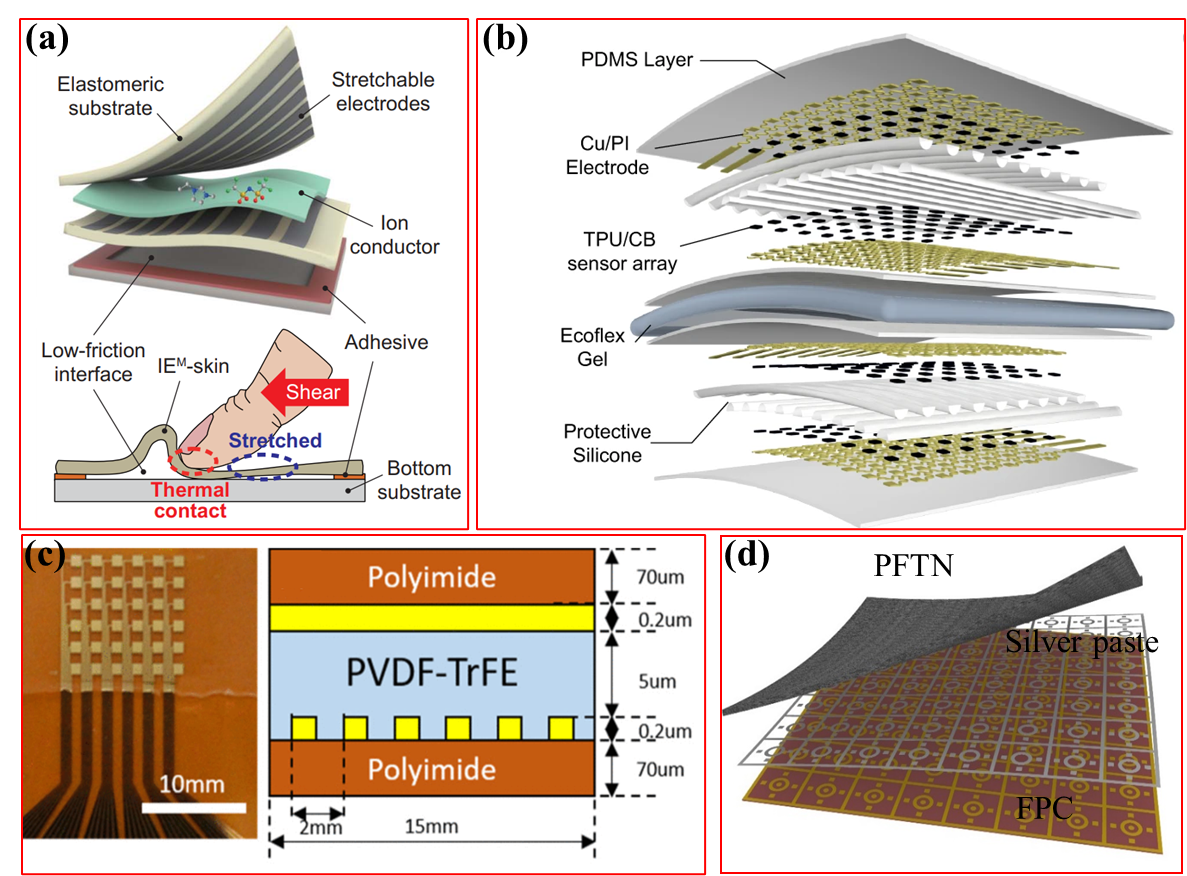


**Figure S13** Comparison between previous researches and our study. (**a**) Artificial multimodal receptors based on ion relaxation dynamics. (**b**) multi-layer piezoresistive array for multi-parameter E-skin. (**c**) High-density piezoelectric sensor array based on Micro-electromechanical Systems (**MEMs)**. (**d**) Our work based on PFTN.


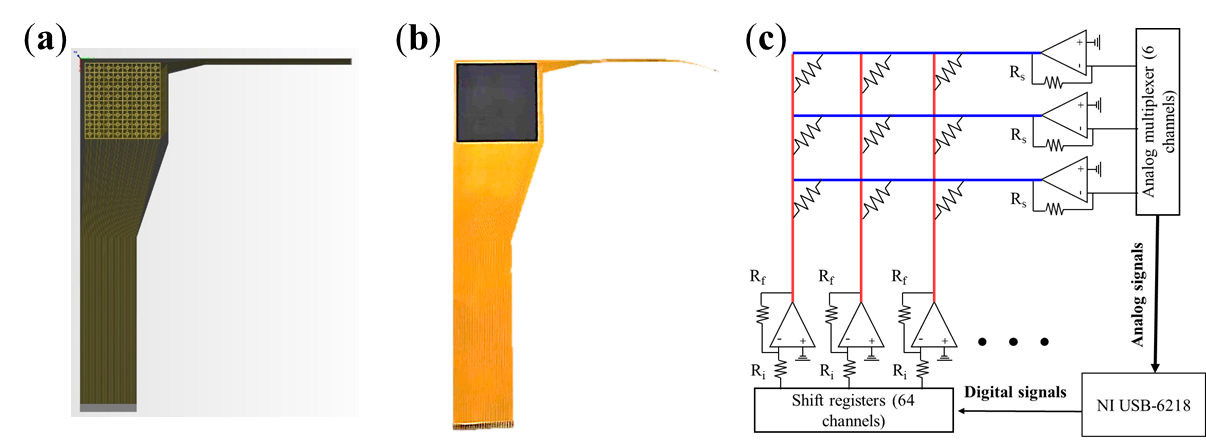


**Figure S14** Structural characteristics of the E-skin and 64×6 channels data acquisition system. (**a**) Schematic view of a FPC supporting the ET-skin. (**b**) Product photo show of the E-skin containing external connection structure. (**c**) electric schematic diagram of the 64×6 channels data acquisition system.


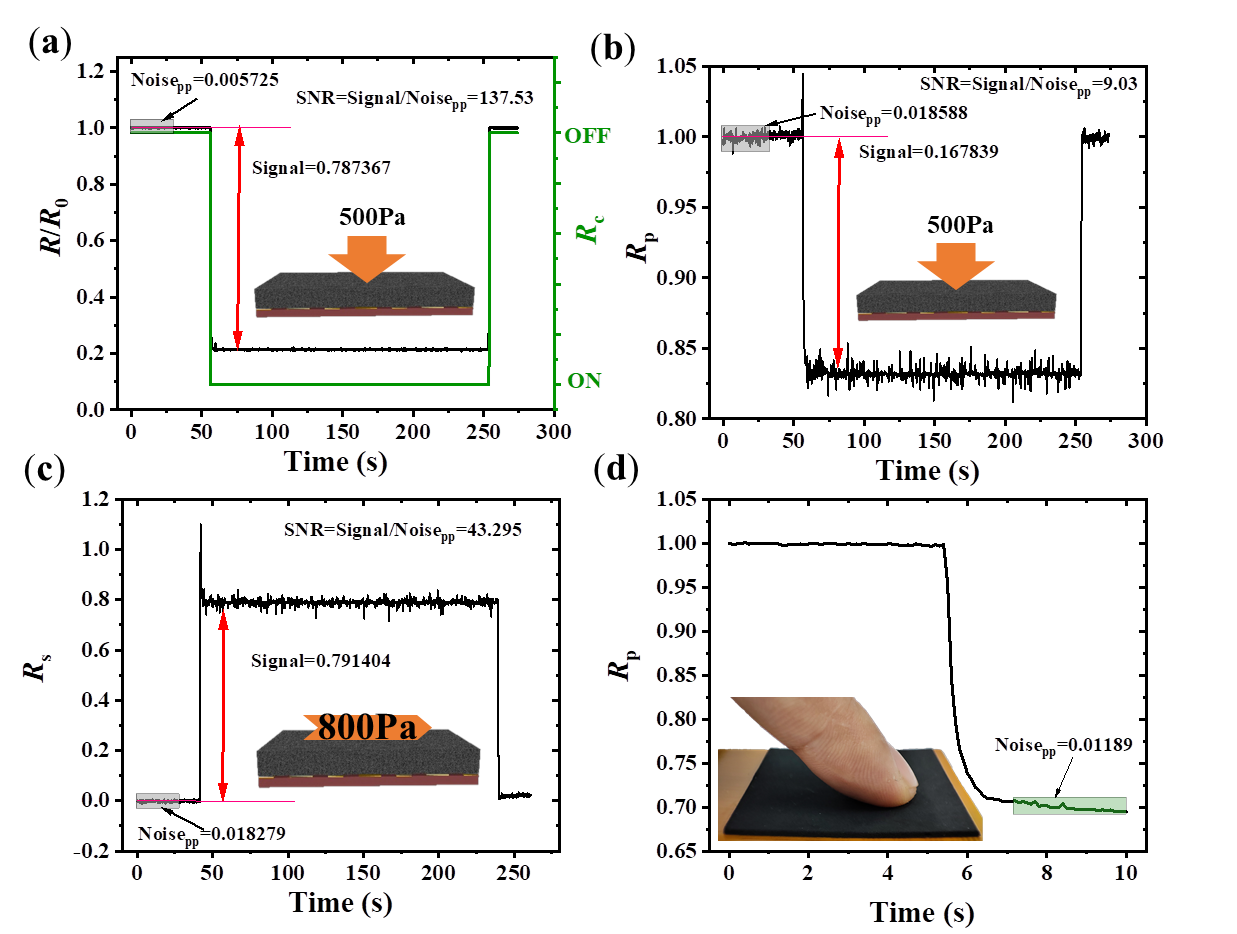


**Figure S15** SNR of E-skin signals. (**a**) SNR information of TEP's contact resistance and the response curve of *R*_c_. (**b**) SNR information of *R*_p_. (**c**) SNR information of *R*_s_. (**d**) *R*_p_ response curve when a finger is pressed on the E-skin.


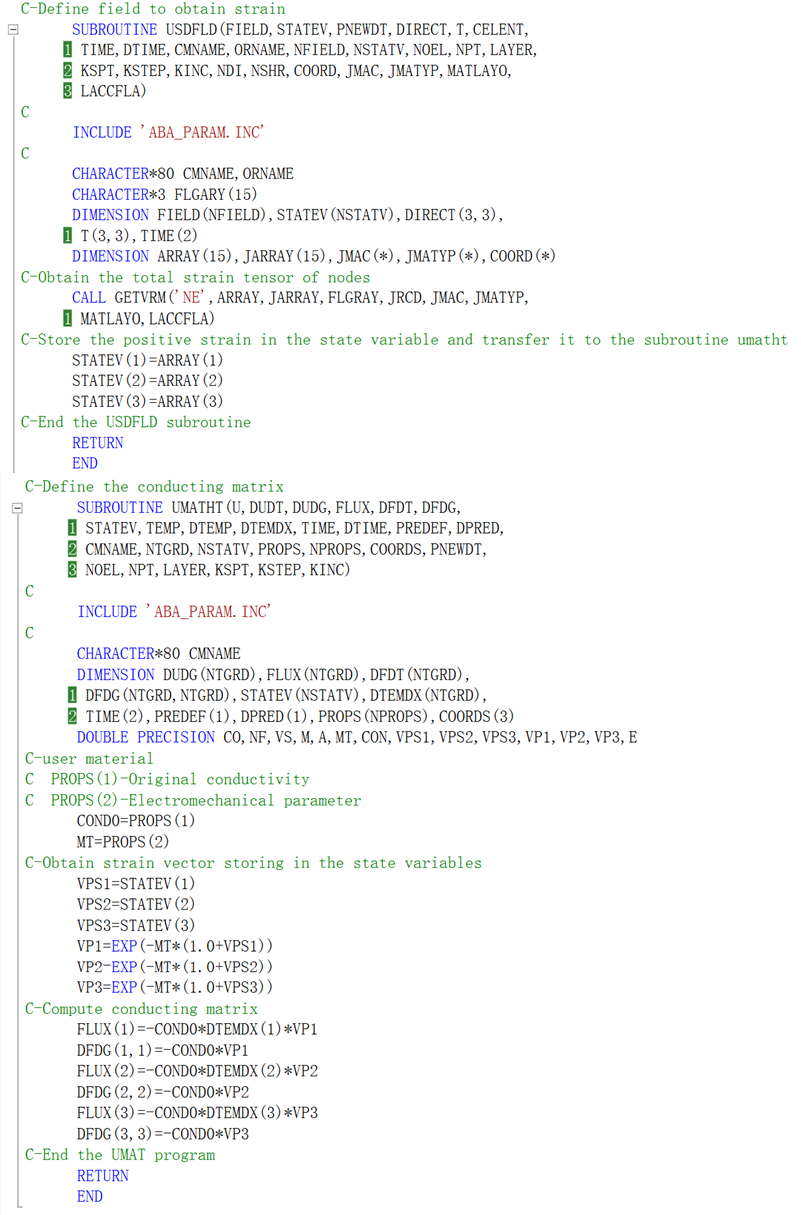


**Figure S16** A subroutine UMAT for FEA by ABAQUS.

**Table S1**. Intrinsic piezoresistive parameters of PFTN with different porosity

| PFTN (12wt%CN) | SPF | P | μ | α | β | *R*_0_ | a | *R*_1_ | b |
| --- | --- | --- | --- | --- | --- | --- | --- | --- | --- |
| 300wt% SN | 50.01% | 0.353 | 83.396 | 4.057 | 0.493 | 0.782 | 0.092 | 0.318 | 1.196 |
| 450wt% SN | 52.11% | 0.331 | 50.424 | 0.765 | 0.756 | 0.973 | 0.571 | 0.027 | -2.457 |
| 600wt% SN | 59.71% | 0.256 | 30.276 | 0.719 | 1.139 | 1.053 | 1.071 | -0.053 | 33.42 |
| 750wt% SN | 64.19% | 0.213 | 14.843 | 0.695 | 1.856 | 1.135 | 1.509 | -0.135 | 14.647 |
| 900wt% SN | 68.23% | 0.179 | 18.421 | 0.255 | 1.129 | 1.070 | 1.112 | -0.07 | 18.675 |

**Table S2**. Intrinsic piezoresistive parameters of PFTN with different content of CN

| **PFTN**  (450wt% SN) | SPF | P | μ | α | β | *R*_0_ | a | *R*_1_ | b |
| --- | --- | --- | --- | --- | --- | --- | --- | --- | --- |
| 8wt% CN | 64.19% | 0.213 | 28.40 | 1.58 | 0.634 | 0.999 | 1.422 | 0.001 | -5.544 |
| 10wt% CN | 64.19% | 0.213 | 28.70 | 0.778 | 1.285 | 0.804 | 1.636 | 0.196 | 1.018 |
| 12wt% CN | 64.19% | 0.213 | 77.062 | 2.52 | 0.397 | 0.779 | 1.785 | 0.321 | 1.021 |
| 15wt% CN | 64.19% | 0.213 | 50.424 | 0.765 | 0.756 | 0.973 | 0.571 | 0.027 | -2.457 |
